# Immune Signatures Identify Patient Subsets Deriving Long‐Term Benefit From First‐Line Rituximab in Follicular Lymphoma

**DOI:** 10.1002/jha2.1103

**Published:** 2025-02-07

**Authors:** Ginevra Lolli, Alessandro Davini, Valentina Tabanelli, Maria Rosaria Sapienza, Federica Melle, Giovanna Motta, Marcello Del Corvo, Angelica Calleri, Anna Vanazzi, Paulina Nierychlewska, Alessio Maria Edoardo Maraglino, Marta Castelli, Maria Chiara Quattrocchi, Roberto Chiarle, Stefano Pileri, Corrado Tarella, Enrico Derenzini

**Affiliations:** ^1^ Oncohematology Division European Institute of Oncology IRCCS Milan Italy; ^2^ Haemolymphopathology Division European Institute of Oncology IRCCS Milan Italy; ^3^ Haematopathology Unit IRCCS Azienda Ospedaliero‐Universitaria of Bologna Bologna Italy; ^4^ Department of Molecular Biotechnology and Health Sciences University of Turin Turin Italy; ^5^ Department of Pathology Boston Children's Hospital Boston Massachusetts USA; ^6^ Department of Health Sciences University of Milan Milan Italy

## Abstract

**Background:**

The role of first‐line single‐agent rituximab immunotherapy in follicular lymphoma (FL) remains debated, as most patients eventually undergo chemotherapy.

**Methods:**

In this study, we retrospectively analyzed 81 FL patients treated with first‐line single‐agent rituximab monotherapy with (*n* = 53) or without (*n* = 28) consolidation. Fifty‐one patients (63%) were high‐tumor burden according to Group d'Etude des Lymphomes Folliculaires (GELF) criteria.

**Results:**

After a median follow‐up of 11 years, overall survival (OS) and progression‐free survival (PFS) rates were 85% and 32%, respectively. Targeted gene expression profiling (T‐GEP) was performed in 40 patients, revealing a 26‐gene expression signature distinguishing complete responders and non‐responders. This signature included genes involved in T‐regulatory (Treg) and natural‐killer cell activity, and interleukin‐17 signaling. A simplified 14‐gene prognostic score (ImSig) enabled accurate outcome stratification in terms of PFS. These data were validated in silico using two independent publicly available cohorts of FL patients treated with chemoimmunotherapy. Deconvolution analyses demonstrated an enrichment in Treg cells in high‐risk ImSig patients, which was validated by immunohistochemistry.

**Conclusions:**

These findings demonstrate that the efficacy of front‐line anti‐CD20 immunotherapy may depend on microenvironment‐related factors, and that specific immune signatures could identify patient subsets obtaining long‐term benefit from a chemo‐free immunotherapeutic approach.

**Trial Registration:**

The authors have confirmed clinical trial registration is not needed for this submission.

## 1 Introduction

Follicular lymphoma (FL) is the most common indolent lymphoma in the United States and in Western Europe [1]. The disease course is usually characterized by a response to initial treatment followed by relapses, sometimes associated with histological transformation into aggressive B‐cell non‐Hodgkin lymphomas (NHL). Despite the improvement in survival rates due to novel therapeutic strategies, FL is still considered a chronic, incurable disease. At diagnosis, most patients have advanced asymptomatic disease [2] and treatment is reserved only for those patients classified as high‐tumor burden [3, 4].

The advent of the anti‐CD20 monoclonal antibody rituximab has revolutionized FL treatment, but the role of first‐line single‐agent rituximab immunotherapy in FL remains debated. Prior trials demonstrated the safety and efficacy of single‐agent rituximab induction followed by consolidation, with a sizeable fraction of patients experiencing long term disease control, including those with high‐tumor burden [5, 6, 7].

However, although some patients may obtain long term benefit with upfront immunotherapy, first‐line standard chemoimmunotherapy offers better results in terms of progression‐free survival (PFS), compared to single‐agent rituximab [7, 8, 9, 10, 11, 12]. Furthermore, first‐line single‐agent rituximab did not demonstrate a significant survival benefit compared to an initial wait and see approach in asymptomatic low‐tumor burden FL [13, 14, 15]. Therefore, immunochemotherapy many times still represents an important step in the first‐line treatment of the vast majority of FL patients in real world practice [4, 14, 15].

In the present study we evaluated the long‐term outcome of a single center cohort of FL patients treated with first‐line single‐agent rituximab. Then, hypothesizing that the efficacy of front line anti‐CD20 in FL could be dependent on microenvironmental factors, we performed targeted gene expression profiling (T‐GEP) using the PanCancer Immune Profiling Panel, to characterize tumor microenvironment (TME) and define immune signatures associated with long‐term outcome.

## 2 Methods

### 2.1 Study Design and Patients

This single‐center retrospective study included all consecutive FL patients treated with rituximab or one of its biosimilars as first‐line monotherapy from 1998 to 2021 at the European Institute of Oncology (IEO) in Milan. Response to treatment, overall survival (OS) and PFS were retrospectively analyzed. Based on the availability of formalin‐fixed paraffin‐embedded (FFPE) tissue from the initial diagnosis (*n* = 40), T‐GEP was performed using the PanCancer Immune Profiling Panel on the NanoString platform. T‐GEP data were correlated with complete remission (CR) rates, PFS, and OS.

All patients signed written informed consent and the study was approved by the institutional review board (protocol no. UID4407) and performed according to the Helsinki Declaration. Response criteria were applied according to the response assessments and restaging procedures of the clinical trials considered in the present study [16, 17, 18] (Table S1). Lugano criteria were applied for patients treated outside clinical trials [18].

### 2.2 T‐GEP and T‐GEP Data Analysis

Profiling of the FL microenvironment was performed on pre‐treatment FFPE tissue in 40 patients using the PanCancer Immune Profiling Panel, which includes 730 genes belonging to the most important immunological pathways, allowing a comprehensive evaluation of the TME.

Five FFPE tissues from reactive lymph nodes were used as negative controls. Detailed information is available in the Supporting Information.

Principal component analysis (PCA) was used for an initial assessment of the gene expression patterns.

Unsupervised hierarchical clustering, with heatmap, and linear regression model were used to evaluate correlations between gene expression levels and response to treatment, which was dichotomized as complete response (CR) and non‐complete response (NCR). The subset of differentially expressed (DE) genes significantly associated with PFS, was then used to construct a Cox proportional hazards model, to define a T‐GEP‐based prognostic score associated with outcome.

The optimal threshold for discriminating patients into two groups at low and high risk was determined using the MaxStat R package.

The T‐GEP‐based prognostic score was validated in silico on two independent, publicly available cohorts of FL patients treated in the first line with immunochemotherapy [19, 20]. Gene expression analyses were performed on FFPE tissue samples using the Affymetrix104 platform in the first cohort [GSE119214] and via NanoString technology (PanCancer Immune Profiling Panel) in the second one [GSE147125].

### 2.3 Deconvolution Analyses

Deconvolution analyses were performed using CIBERSORTx [21], which allowed the calculation of the relative cellular fractions of 22 immune cell types in the TME. Detailed description of deconvolution analyses is available in Supporting Information.

### 2.4 Immunohistochemistry

Twenty‐nine cases were selected for immunohistochemical analysis based on the availability of high‐quality biopsy tissue. Images were then analyzed using the open‐source software “QuPath” (version 0.5.0) [22], to generate single‐cell detections and to assess the percentage of forkhead box P3 (FOXP3) positive cells. Additional information on image analysis is available in the Supporting Information.

### 2.5 Statistical Analyses

OS was calculated from start of treatment to the date of death due to any cause, and was censored at the last date the patient was known to be alive. PFS was calculated at the time from the beginning of treatment until lymphoma progression or death due to any cause.

Survival curves were created with the Kaplan–Meier method [23]; differences between groups were calculated using the log rank test.

## 3 Results

### 3.1 Patients' Characteristics

**FIGURE 1:**
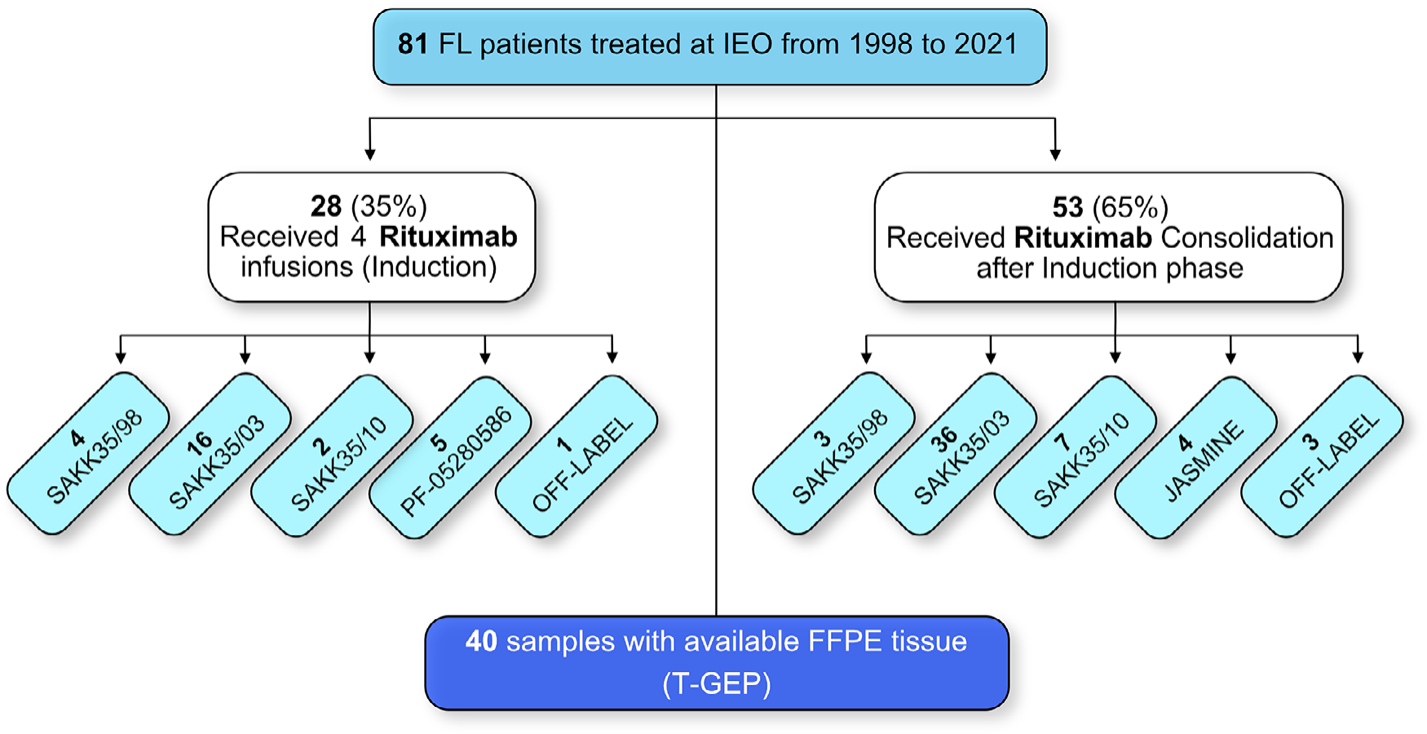
Consort diagram describing the study flow. The 81 consecutive follicular lymphoma patients treated at IEO are divided into those who received four rituximab infusions (induction phase) and those who received rituximab consolidation after induction (consolidation phase). The clinical trials in which the patients were enrolled are indicated.

Eighty‐one consecutive patients over 18 years of age, with a confirmed histological diagnosis of FL, treated with first‐line single‐agent anti‐CD20 immunotherapy (rituximab or biosimilars) from 1998 to 2021 were included in this study (Figure 1). Median age at the time of diagnosis was 55 years (range: 25–80). Fifty‐one patients (63%) were classified as high‐tumor burden according to the GELF criteria. Seventy‐eight (96%) patients were treated in the context of clinical trials and four patients were treated with off‐label rituximab [7, 24–28]. Patients' characteristics are described in Table 1 and clinical trials in Table 2.

**TABLE 1:** Patients' characteristics in the whole population and in the T‐GEP cohort.

Patients' characteristics	Total *N* = 81	Total *N* = 40	*p* value (FDR‐correction)
Sex			
Male *n* (%)	39 (48)	18 (45)	1
Female *n* (%)	42 (52)	22 (55)	
Median age (range)	55 (25–80)	55 (35–80)	1
Stage			
I/II *n* (%)	23 (28)	18 (45)	0.5
III/IV *n* (%)	58 (72)	22 (55)	
FLIPI^a^			
0–2 (low‐intermediate) *n* (%)	62 (77)	32 (80)	1
3–5 (high) *n* (%)	17 (21)	7 (18)	
GELF criteria^b^			
Present *n* (%)	51 (63)	27 (67)	1
Absent *n* (%)	29 (36)	13 (33)	

^a^ For two patients it was not possible to evaluate FLIPI score.

^b^ For one patient it was not possible to evaluate the presence of the criteria at the time of diagnosis.

**TABLE 2:** Different patients' rituximab treatment schedules according to the respective clinical trials.

Clinical study	No. of patients (%)	Rituximab treatment schedule
*SAKK 35/98*	7 (9)	Induction therapy: 4 weekly doses Then patients are randomized to observation (*N* = 4) or to 4 additional maintenance doses every 2 months (*N* = 3)
*SAKK 35/03*	52 (64)	Induction therapy: 4 weekly doses Then patients without exclusion criteria and with at least a partial response (PR) (*N* = 34) were randomized to four additional doses every 2 months (*N* = 21) or to maintenance administration every 2 months for up to 5 years (*N* = 21) 15 patients did not receive maintenance therapy
*SAKK 35/10*	9 (12)	Induction therapy: 4 weekly doses Then four maintenance doses from Weeks 12 to 15
*PF‐05280586*	5 (6)	Induction therapy: 4 weekly doses
*JASMINE*	4 (5)	Induction therapy: 4 weekly doses Then two maintenance doses at Weeks 12 and 20
*OFF‐LABEL R*	4 (5)	Induction therapy: 4 weekly doses Then two maintenance doses at Weeks 8 and 12 (*N* = 1) or 4 monthly maintenance doses (*N* = 2) One patient did not receive maintenance therapy

FFPE tissue collected before the start of treatment was available in 40 patients (T‐GEP cohort) (Figure 1). Characteristics of patients in the T‐GEP cohort were similar compared to the whole cohort (Table 1).

### 3.2 Response to Induction Therapy and Long‐Term Outcome

#### 3.2.1 Whole Cohort

**FIGURE 2:**
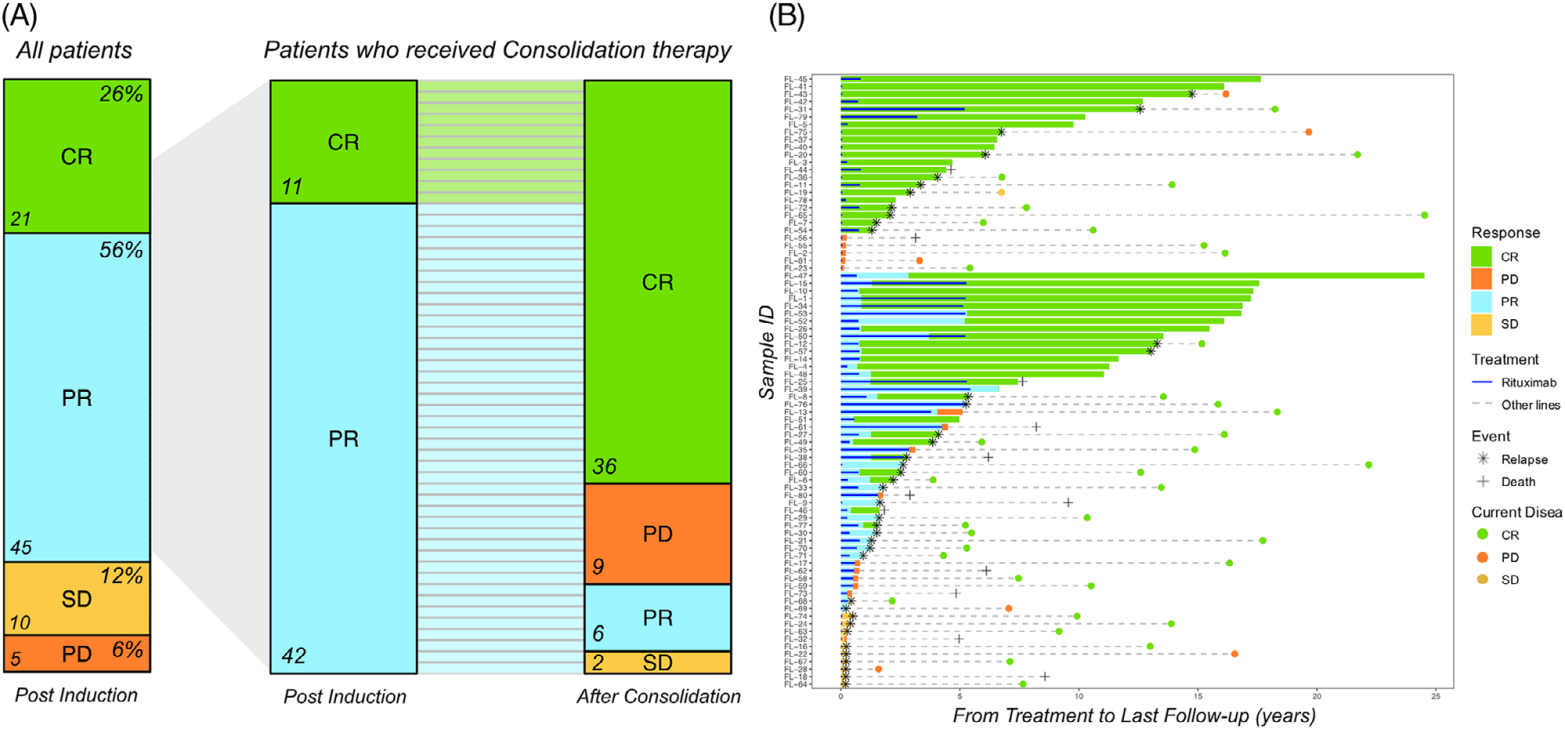
(A) Post‐induction response for all the 81 patients; response conversion between induction phase and consolidation phase for the 53 patients who received rituximab consolidation. (B) Swimmer plot describing the response to the treatment over time for the entire cohort of 81 patients.

All patients considered in the present study were evaluable for response. The ORR at the end of the induction treatment was 81% (*n* = 66) (Figure 2A), with 21 patients (26%) in CR and 45 patients (56%) in PR (Figure 2A). Among patients who responded to induction treatment, 38 patients (58%) met the GELF criteria [13] at rituximab start, while 27 (41%) did not. Fifty‐three patients (65%) responding to induction therapy (of whom 42 in PR and 11 in CR), received consolidation therapy: 25 of 42 patients (60%) converted from PR to CR during or after consolidation (Figure 2A). Median time of conversion from PR to CR was 8 months (range: 1–60).

**FIGURE 3:**
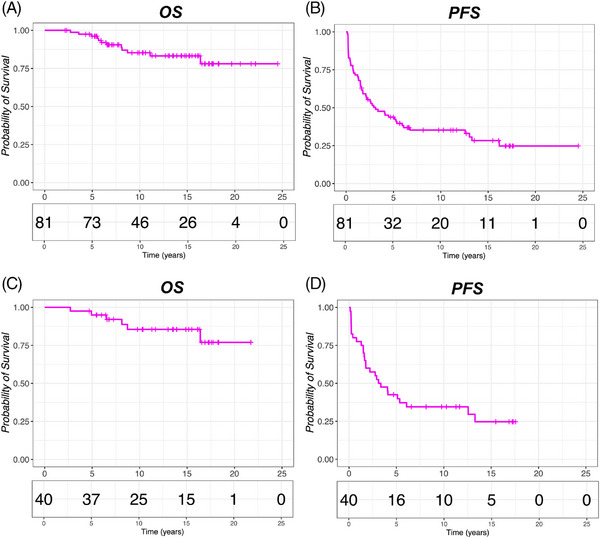
(A, B) Overall survival (OS) and progression‐free survival (PFS) curves generated for the entire cohort of 81 patients. (C, D) OS and PFS curves for the T‐GEP cohort (*n* = 40).

After a median follow‐up of 11 years, 26 patients (32%) are in continuous CR while the remaining 55 (68%) have experienced disease relapse or progression (Figure 2B). Notably, 42% (*n* = 11) of long‐term complete responders were high‐tumor burden. The 10‐year PFS and OS rates of the whole cohort are 35% and 85% (Figure 3A,B).

During the study observation period 15 patients (19%) developed secondary neoplasms, and 12 deaths (15%) were recorded at the last follow‐up. Detailed information is available in the Supporting Information.

#### 3.2.2 T‐GEP Cohort

Forty patients had FFPE tissue from initial diagnosis available for T‐GEP studies (T‐GEP cohort). Of those, 32 patients (80%) responded to the induction treatment obtaining a PR (*n* = 22, 55%) or a CR (*n* = 10, 25%). PFS and OS rates of the T‐GEP cohort were superimposable to the whole cohort (Figure 3C,D). Detailed information is available in Figure S1.

Twenty‐six patients (65%) who achieved CR (*n* = 5) or PR (*n* = 21) after induction therapy, received further consolidation administrations. At the end of the complete induction plus consolidation treatment, 23 patients (58%) achieved a CR, 5 (12%) were in PR, 5 (12%) had SD, and 7 (18%) were in PD (Figure S1). Thirty‐four (85%) out of the 40 patients were alive at the last follow‐up. After a median follow‐up of 11 years, 12 patients (30%) are in continuous CR while the remaining 28 (70%) had experienced disease relapse or PD.

### 3.3 Impact of Clinical Variables on Disease Outcome Following Single‐Agent Rituximab Immunotherapy

We analyzed the impact of established clinical variables (presence or absence of GELF criteria, consolidation therapy, POD24, Follicular Lymphoma International Prognostic Index score [FLIPI] score [0–2 vs. 3–5]) on PFS and OS (Figure S2). Among these variables, only FLIPI score correlated with OS in univariate analyses. Regarding PFS, rituximab consolidation therapy, absence of GELF criteria, and FLIPI 0–2 were associated with a more favorable outcome in univariate analyses (Figure S2A).

Focusing the analyses to the T‐GEP cohort (*n* = 40) we observed similar findings, with the exception of GELF criteria and FLIPI score which were not significantly associated with PFS (Figure S2B).

### 3.4 T‐GEP Immune Signatures Are Associated With Outcome Following First‐Line Single‐Agent Rituximab Immunotherapy in FL

Hypothesizing that the efficacy of first‐line single‐agent rituximab immunotherapy could be influenced by the TME, we investigated associations between expression levels of 730 genes involved in cancer immune processes (PanCancer Immune Profiling Panel) and response to the complete treatment plan (rituximab induction alone or rituximab induction + consolidation).

T‐GEP was performed in 40 patients with available FFPE tissue from lymph node biopsies performed before the start of first‐line rituximab therapy.

**FIGURE 4:**
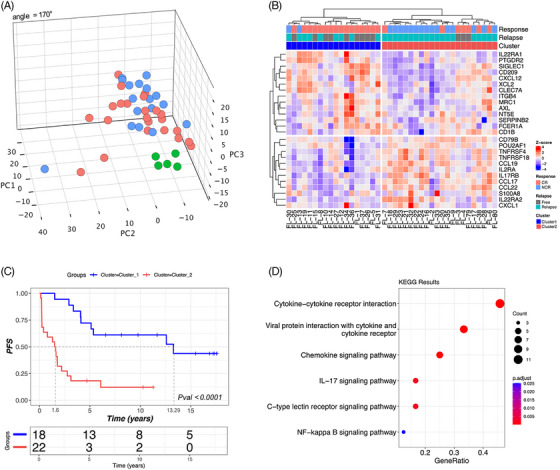
(A) Principal component analysis (PCA) showing a good separation between FL patients (*n* = 40) and negative control samples (*n* = 5). (B) Unsupervised heatmap generated on the 26‐gene signature identifying two clusters significantly associated with complete response outcomes. (C) PFS curves generated for the two identified clusters. Median PFS is 13.3 years for patients in Cluster 1 versus 1.6 years in Cluster 2 (*p* < 0.0001) [4]. Pathway analysis showing a significant association between the 26‐gene signature and the activity of T‐regulatory (Treg) cells, natural killer (NK) cells, and cytokine‐chemokine signaling, including the interleukin‐17 signaling pathway.

PCA showed a good separation between FL patients (*n* = 40) and the negative control samples (*n* = 5 reactive follicular hyperplasia) (Figure 4A).

We identified 26 genes significantly associated with outcome in terms of CR at the end of the complete treatment plan (Figure 4B). In particular, unsupervised clustering identified two patient subsets: CR rate at the end of therapy was 89% in Cluster 1 versus 33% in Cluster 2 (*p* < 0.0001), and median PFS was 13.3 years for patients in Cluster 1 versus 1.6 years for patients in Cluster 2 (*p* < 0.0001) (Figure 4C).

**FIGURE 5:**
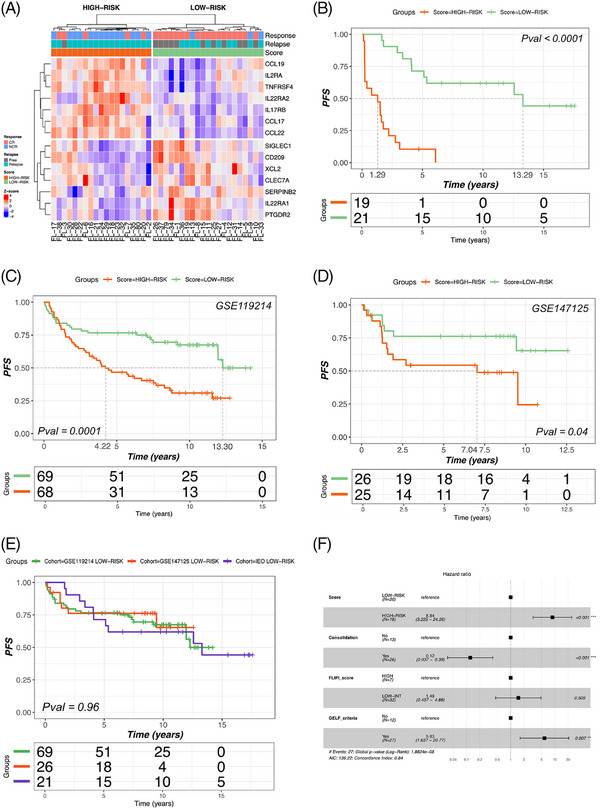
(A) Heatmap generated from the ImSig showing the stratification of patients into two groups at low and high risk of treatment failure. (B) PFS curves generated for the two groups. Median PFS is 1.3 years for high‐risk patients versus 13.3 years for low‐risk patients (*p* < 0.0001). (C) PFS curve generated for a publicly available cohort of 137 FL patients treated with first‐line immunochemotherapy. (D) PFS curve generated for a publicly available cohort of 51 FL patients treated with first‐line immunochemotherapy. (E) Low‐risk patients in the validation cohorts and low‐risk patients in our discovery cohort show superimposable PFS curves. (F) Multivariate framework confirming ImSig as an independent prognostic predictor, stratifying low‐ and high‐risk patients regardless of other clinical variables.

Pathway analysis showed a significant association between DE genes and activity of T‐regulatory (Treg) cells, natural killer (NK) cells, and cytokine‐chemokine signaling, including the interleukin‐17 signaling pathway (Figure 4D). Next, we constructed a simplified 14‐gene prognostic risk model for outcome prediction, here after defined as ImmunoSignature (ImSig) (Figure 5A).

The 14 genes defining the ImSig are: *IL2RA*, *IL22RA2*, *IL22RA1*, *CCL17*, *CCL19*, *CCL22*, *SIGLEC1*, *PTGDR2*, *XCL2*, *CLEC7A*, *TNFRSF4*, *IL17RB*, *SERPINB2*, and *CD209*. The overexpression of *IL22RA2*, *CCL22*, *TNFRSF4*, *IL17RB*, *IL2RA*, *CCL17*, *CCL19*, and the downregulation of *CD209*, *SIGLEC1*, *XCL2*, *CLEC7A*, *SERPINB2*, *ILL22RA1*, *PTGDR2* were significantly associated with a worse prognosis.

The ImSig allowed stratification of patients into two groups at low and high risk of treatment failure. In our cohort, 19 (48%) patients were classified as high‐risk according to the ImSig, while 21 (52%) were classified as low‐risk.

As shown in Figure 5B, ImSig was a powerful outcome predictor, identifying a subset of patients deriving durable benefit from first‐line rituximab monotherapy: in fact, median PFS was 1.3 years for high‐risk versus 13.3 years for low‐risk patients. Notably, the clinical characteristics of patients belonging to the high‐ and low‐risk groups according to ImSig were similar, with respect to FLIPI score, GELF criteria, and fraction of patients receiving consolidation therapy (Table 3).

**TABLE 3:** Patients' characteristics in the two groups: High‐risk ImSig and low‐risk ImSig.

Patients' characteristics	Low‐risk (ImSig) *N* = 21 (%)	High‐risk (ImSig) *N* = 19 (%)	*p* value (FDR‐correction)
Sex			
Male *n* (%)	11 (52)	7 (37)	0.75
Female *n* (%)	10 (48)	12 (63)	
Median age (range)	50 (35–78)	57 (41–80)	0.3
Stage			
I–II *n* (%)	9 (43)	9 (47)	1
III–IV *n* (%)	12 (57)	10 (53)	
FLIPI			
0–2 (low‐intermediate risk) *n* (%)	17 (81)	15 (79)	1
3–5 (high risk) *n* (%)	3 (14)	4 (21)	
GELF criteria			
Present *n* (%)	13 (62)	14 (74)	0.75
Absent *n* (%)	8 (38)	5 (26)	
Consolidation			
Yes *n* (%)	17 (81)	9 (47)	0.3
No *n* (%)	4 (19)	10 (53)	

Multivariate framework confirmed that ImSig was an independent prognostic predictor, stratifying low‐ and high‐risk patient subsets irrespective of other clinical variables (Figure 5F).

The prognostic value of ImSig was tested on two independent, publicly available cohorts of FL patients treated with chemoimmunotherapy [19, 20]. ImSig allowed accurate prognostic stratification in both cohorts, with low‐risk ImSig patients having a significantly better outcome compared to high‐risk patients. Importantly, 5‐ and 10‐year PFS rates of low‐risk patients treated with chemoimmunotherapy in the validation cohorts mirrored the results of low‐risk patients treated with single‐agent immunotherapy in our discovery cohort (Figure 5C–E).

### 3.5 A TME Enriched in Treg Cells Defines High‐Risk FL Patients' Subsets Following First‐Line Single‐Agent Rituximab Monotherapy

**FIGURE 6:**
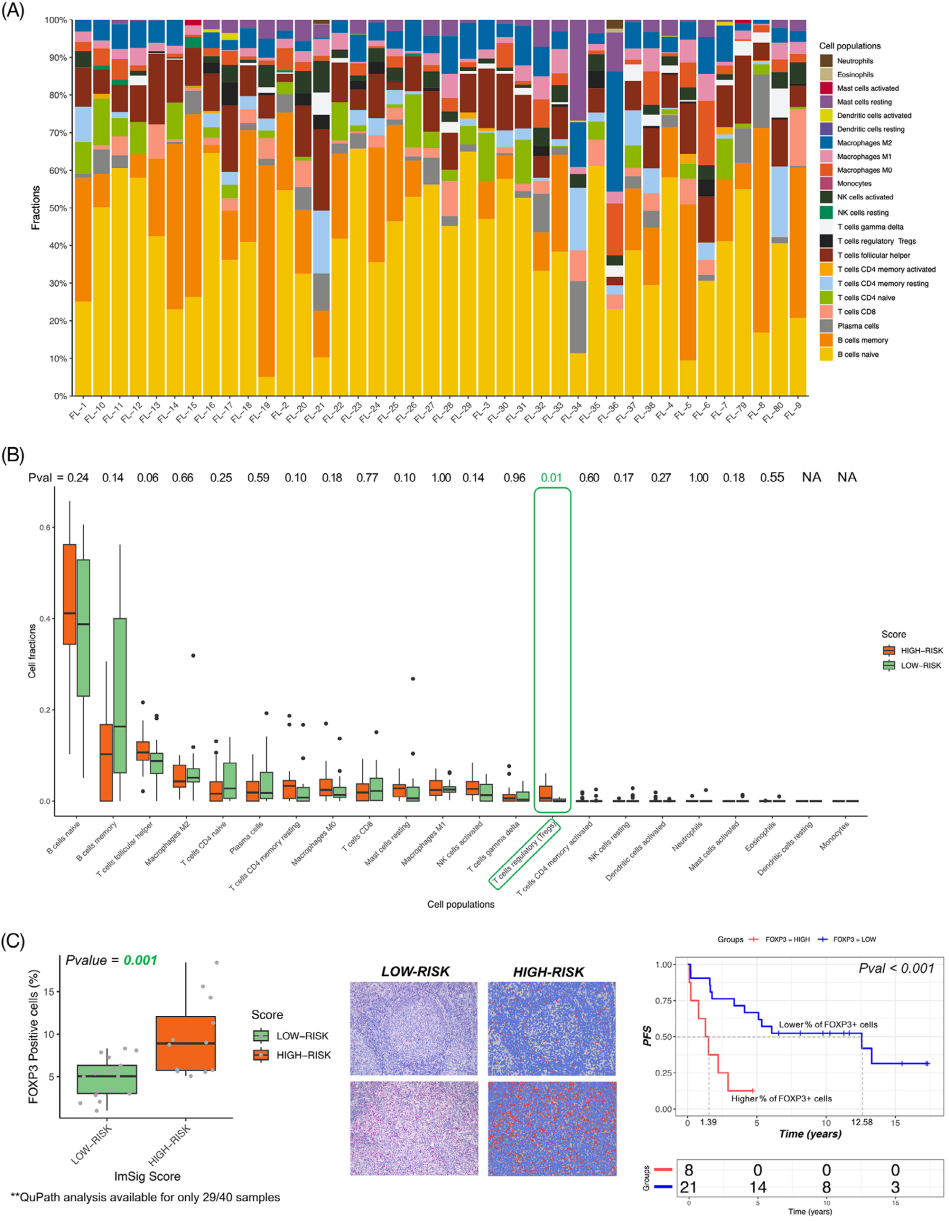
(A) CIBERSORTx deconvolution analyses showing the cellular composition of TME from bulk GEP data. (B) Comparison of the cellular composition between high‐ and low‐risk patients with Treg cells significantly more represented in the high‐risk group of patients. (C) Immunohistochemistry results show that high‐risk ImSig patients are characterized by a significantly higher fraction of FOXP3‐positive cells. Representative immunostaining for FOXP3 in brightfield (middle‐left) and corresponding cell detections are illustrated as nuclear overlays; FOXP3‐positive cells are color‐coded in red (middle‐right). Higher levels of Treg cells are associated with worse outcome in terms of PFS.

To investigate the implications of ImSig and its impact on the composition of the TME, we performed deconvolution analyses of our T‐GEP data using CIBERSORTx [21]. Patients classified as low‐ and high‐risk categories according to ImSig did not show remarkable differences in terms of cellular fractions, with exception for Treg cells, which were significantly more represented in the high‐risk group (Figure 6A).

To validate deconvolution data, we performed immunohistochemistry staining, assessing expression levels of transcription factor FOXP3 on T lymphocytes. In fact, FOXP3 is a well‐established marker of Treg cells, as its sustained expression is indispensable for their phenotypic stability, metabolic fitness, and regulatory function [29].

Twenty‐nine of the 40 samples considered in T‐GEP analyses were available for FOXP3 immunohistochemistry assessment. In line with T‐GEP data, we found that high‐risk ImSig patients were characterized by a significantly higher fraction of FOXP3 positive cells. Importantly, higher levels of Treg cells, indicative of an immunosuppressive microenvironment, were associated with worse outcome in terms of PFS (Figure 6B,C).

## 4 Discussion

In the present study we describe the long‐term outcome of a single center cohort of FL patients treated with first‐line rituximab monotherapy, confirming that a sizeable fraction of patients (about 30%) may achieve long‐term remission [7, 24–28], and demonstrating that the characterization of TME by T‐GEP and/or immunohistochemistry could identify patients' subsets deriving maximal benefit from a first‐line chemo‐free immunotherapeutic strategy. Of note among patients who responded to induction treatment, 58% of patients (*n* = 38) met the GELF criteria at the start of rituximab therapy and 42% (*n* = 11) of long‐term complete responders were high‐tumor burden, indicating that long‐term disease control can be achieved with single‐agent rituximab in a patient population that would be otherwise treated with standard chemoimmunotherapy.

In line with prior observations [30, 31] and with a long median follow‐up (11 years), these data confirm that the omission of chemotherapy as first‐line treatment can be considered a safe treatment strategy with no detrimental impact on OS (80% of patients considered in the present study may be considered as long‐term survivors). Interestingly, POD24 did not affect OS rates in this study, suggesting that the prognostic value of early relapse could be limited to patients treated with chemoimmunotherapy.

Regarding consolidation therapy, an important fraction of patients converted from PR to CR during consolidation, and CR conversion was observed by Cycle 4 in most instances. Accordingly, patients receiving rituximab consolidation therapy had a more favorable PFS compared to patients treated only with four induction cycles, in line with SAKK studies [24, 25, 26, 27].

Given that the main mechanism of action of rituximab is antibody‐dependent cytotoxicity through the engagement of immune effector cells, we hypothesized that a molecular characterization of FL microenvironment could provide valuable information for patient stratification and outcome prediction. For this purpose, we took advantage of our single center cohort and profiled those cases with available FFPE tissue biopsies (*n* = 40) from initial diagnosis with T‐GEP, applying the PanCancer Immune Profiling Panel on the NanoString platform. We defined a gene expression‐based risk score (ImSig), which was significantly associated with PFS, successfully stratifying patients in subgroups at low‐ and high‐risk of treatment failure following first‐line single‐agent rituximab immunotherapy. Notably, the median PFS was 1.3 and 13.3 years for high‐ and low‐risk patients, respectively. Importantly, multivariate analyses demonstrated that the prognostic power of ImSig was independent from established clinical variables, such as consolidation therapy, GELF criteria, and FLIPI score. We think that the similarity in outcomes and clinical characteristics between the unselected series of 81 consecutive FL patients and the smaller T‐GEP cohort supports the validity and generalizability of the data presented here.

Due to the lack of publicly available datasets of patients treated with single‐agent rituximab as first‐line therapy, we decided to validate ImSig in silico, using two independent datasets of 137 and 51 patients treated with first‐line chemoimmunotherapy. Interestingly, with all the limitations of an indirect comparison, the outcome of low‐risk ImSig patients in our first‐line rituximab cohort was superimposable to the outcome of low‐risk ImSig patients in the in silico chemoimmunotherapy validation cohorts. These data suggest that ImSig could identify a subset of patients deriving maximal benefit from single‐agent rituximab and who could be potentially spared from chemotherapy and its related toxicities. By performing deconvolution analyses, we demonstrated that high‐risk ImSig patients are characterized by an immunosuppressive microenvironment enriched in Treg cells. This finding was confirmed by immunohistochemistry, with high‐risk ImSig patients showing higher fraction of FOXP3 positive Treg cells in the TME. Our data could suggest that FOXP3 staining serves as a potential biomarker for identifying patients at high‐risk of treatment failure and confirms early studies on the role of FOXP3 as a prognostic factor in FL [32].

The role of TME in FL has been extensively investigated by several studies with quite heterogeneous results on the prognostic role of tumor‐infiltrating and systemic T‐cells, possibly due to the diverse methodologies and disparate treatments received by FL patients considered in different studies [33, 34, 35, 36]. The relevance of TME characterization has been underlined also by two recent studies based on single‐cell RNA sequencing [37, 38].

These studies highlighted different aspects of TME in FL, distinguishing several FL subtypes according to the TME composition, and shedding light on intratumor heterogeneity and important immune escape mechanisms which could be target for tailored therapeutic interventions. Taken together, these data confirm the complexity of the FL microenvironment and suggest that the prognostic role of TME in FL could be context‐dependent and potentially influenced by the specific therapeutic regimen administered.

Thanks to the advent of an increasing number of targeted immunotherapies, we are moving toward a chemotherapy‐free approach in FL [2, 39–47]. The RELEVANCE trial demonstrated that a treatment with rituximab and lenalidomide (R2) mirrors the efficacy of standard chemoimmunotherapy [48, 49]. More recently, preliminary results of anti‐CD20 bispecific antibodies as first‐line FL treatment are very encouraging and clinical trials are underway [50, 51, 52].

The present study has some limitations. First of all, the relatively small patient population with available FFPE tissue for T‐GEP analyses (*n* = 40 patients) which makes it difficult to perform stratified OS analyses, due to the paucity of events. Moreover, patients included in the present study were enrolled in different clinical trials and treated in a time frame of more than 20 years, which could introduce possible confounding factors.

However, as far as we know, there are no established biomarkers allowing the a priori identification of patients who would benefit most from single‐agent anti‐CD20 immunotherapy in FL. Our data suggest that low‐risk ImSig (low FOXP3) FL patients could derive long‐term benefit from single‐agent rituximab, suggesting that loss of immune surveillance could be an important mechanism influencing patient's outcome and underlying therapy resistance in FL.

In conclusion, in a clinical scenario where multiple chemo‐free options will be available in the next future, our study could provide the rationale for biomarker‐driven clinical studies in FL patients treated with chemo‐free treatment strategies.

## Author Contributions

G.L. and A.D. designed the study, acquired and analyzed patients' data, wrote the manuscript. V.T. performed immunohistochemistry and helped in writing the manuscript. M.R.S., F.M., and G.M. performed targeted gene expression profiling. M.D.C. helped with bioinformatics analysis. A.C. performed immunohistochemistry. A.V., P.N., A.M.M., M.C., and M.C.Q. helped with patients' data acquisition and analysis. R.C., S.P., and C.T. helped with data interpretation and critically revised the manuscript. E.D. conceptualized the research, designed research studies, wrote the manuscript.

## Conflicts of Interest

Enrico Derenzini: Research funding: Takeda, ADC‐Therapeutics, Incyte; Speaker's bureau: Roche, Incyte, Abbvie; Advisory Board: Astra Zeneca, Lilly, Abbvie, Roche, Gilead, Takeda, Sobi. Stefano Pileri: Speaker's bureau: Lilly, Takeda, BeiGene, Stemline, Roche; Advisory Board: Lilly, Stemline, Diatech. The other authors declare no conflicts of interest.

## Supporting information



Supporting Information

Supporting Information

Supporting Information

## Data Availability

Normalized T‐GEP with associated outcome information can be found in the Supporting Information.
