# Supplementary figures and images for: Immune Signatures Identify Patient Subsets Deriving Long‐Term Benefit From First‐Line Rituximab in Follicular Lymphoma
Source: EJHaem. 2025 Feb 7;6(1):e1103. doi: 10.1002/jha2.1103 (PMC11804214; doi:10.1002/jha2.1103)

A

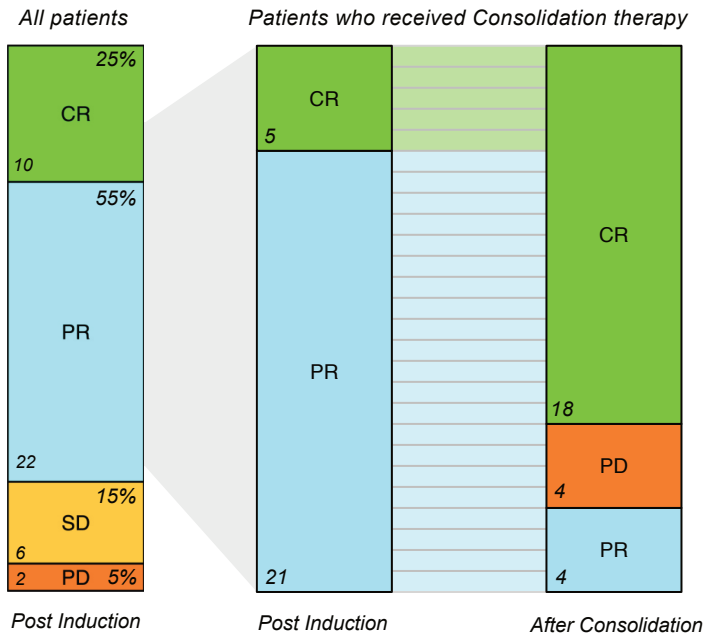

B

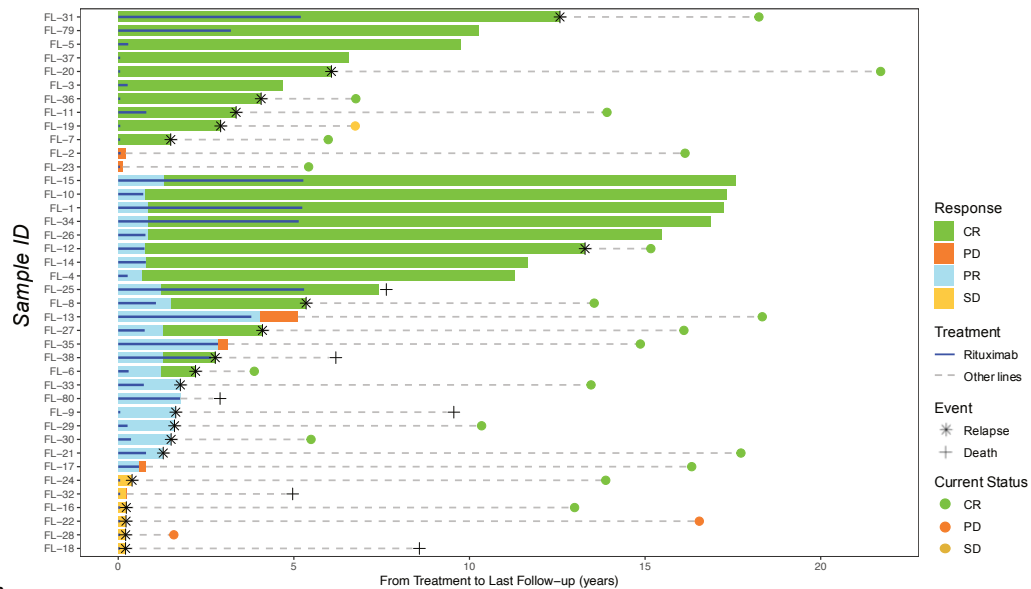

Figure S1

A

N= 81

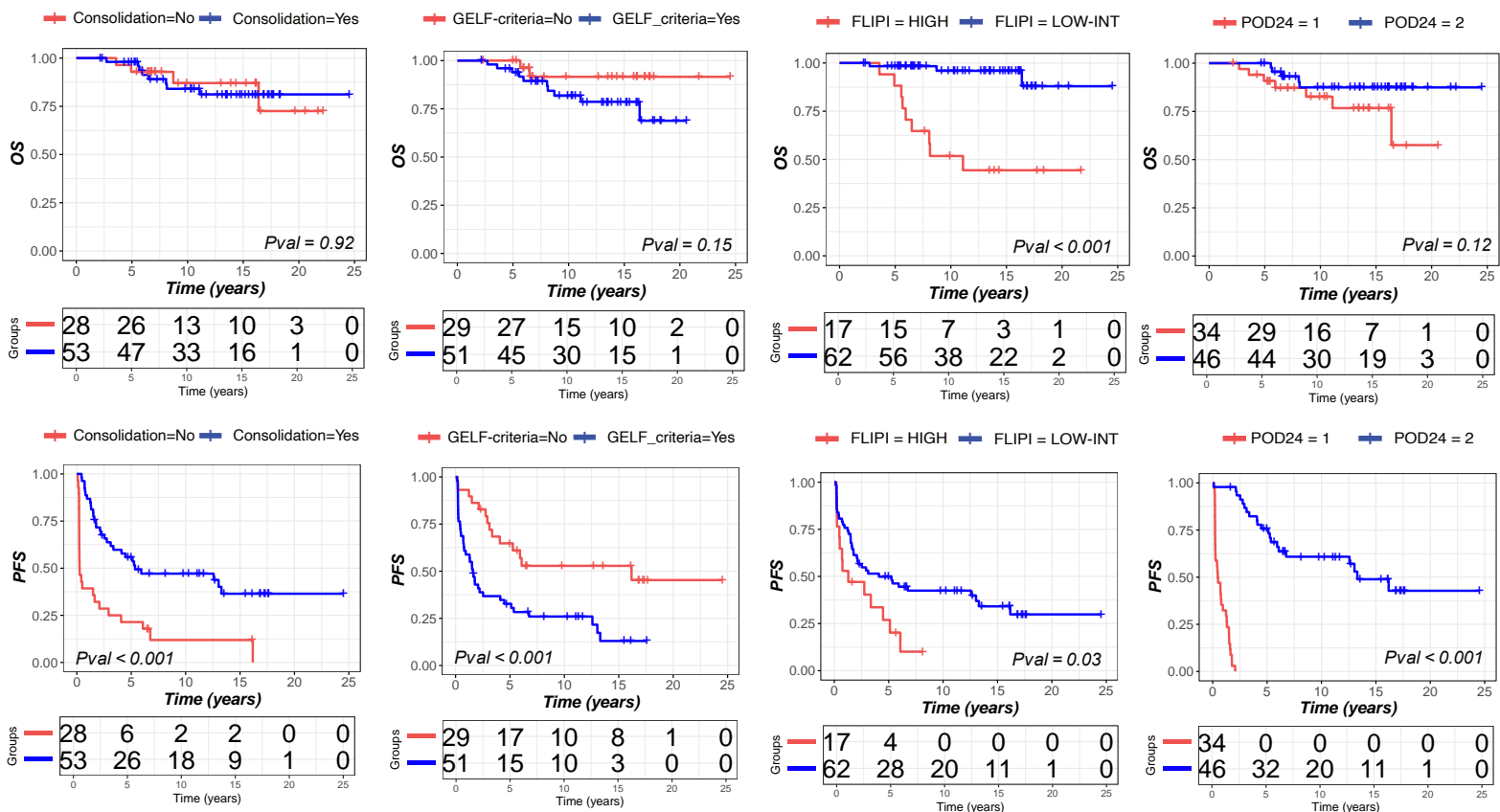

B

N= 40

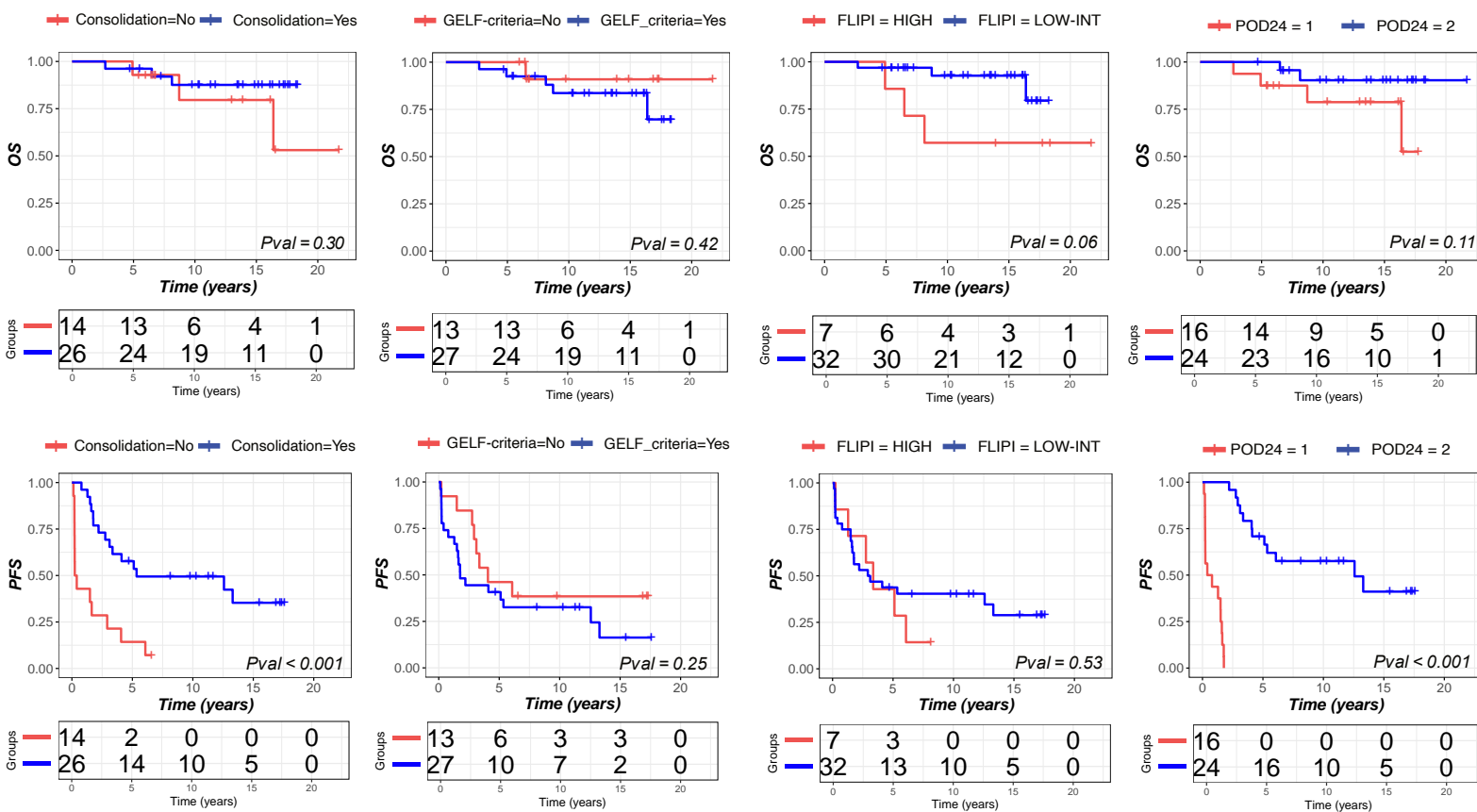

Figure S2

Supplement: Supplementary file 1 — Supporting Information [file JHA2-6-e1103-s001.pdf]
