# Supplementary material for: Immune Signatures Identify Patient Subsets Deriving Long‐Term Benefit From First‐Line Rituximab in Follicular Lymphoma
Source: EJHaem. 2025 Feb 7;6(1):e1103. doi: 10.1002/jha2.1103 (PMC11804214; doi:10.1002/jha2.1103)
Supplement: Supplementary file 2 — Supporting Information [file JHA2-6-e1103-s003.docx]

**Supplemental information**

**Causes of death and long-term toxicities**

At the last follow-up there were 12 deaths (15%). Six patients died with relapsed/refractory disease: 4 deaths (33% of all deaths) were due to disease recurrence (1 due to histological transformation of FL into DLBCL), 2 deaths (17%) occurred following a diagnosis of secondary acute myeloid leukemia (AML),

Six patients died in continuous CR: one patient for chronic obstructive pulmonary disease, another patient for primary bone cancer, and for the remaining 4 patients the cause of death was unknown.

Overall, the cause of death was directly attributable to FL progression or transformation in 4 patients (5%).

During the follow-up, 15 patients (19%) had secondary neoplasms. The following diagnosis of cancer were recorded: 1 patient had a renal cell carcinoma, 1 patient a typical lung carcinoid, 3 patients were diagnosed with melanomas (one with also marginal NHL), 1 patient with a primary bone tumor, 1 patient was diagnosed with endotracheal squamous cell carcinoma, lung squamous cell carcinoma and colon adenocarcinoma. Furthermore, 1 patient was diagnosed with marginal NHL and 1 with mantle cell lymphoma (confirmed by histological examination). 3 patients were diagnosed with secondary acute myeloid leukemia (AML); 1 of these 3 patients underwent histological transformation to aggressive lymphoma (DLBCL) and subsequently to AML. Finally, 1 patient was diagnosed with basal cell carcinoma, 1 with colon adenocarcinoma and 1 with urothelial carcinoma.

**Supplemental Materials and Methods**

**Target gene expression profiling (T-GEP)**

Messenger RNA (mRNA) was extracted from FFPE tissue from nodal biopsies performed before the start of first-line rituximab treatment using the Recover All Total Nucleic Acid Isolation Kit and its quality was defined based on the value of DV200 (percentage of RNA fragments with ≥200 nucleotides). The mRNA expression levels of target genes​​ were measured using the NanoString nCounter Analysis platform. The system calculates the relative abundance of each mRNA transcript of interest through a hybridization assay. The mRNA is first hybridized with pairs of probes (Reporter Probe and Capture Probe) specific for each target of interest and, after a purification step, during which the excess of unbound probes is removed, the tripartite target-target complex probes are immobilized on the surface of a cartridge which is then scanned. Each fluorescent barcode, linked to the reporter probe, is counted. The hybridized samples are then loaded into the nCounter Prep Station for post-hybridization processing. Quality control and normalization of NanoString nCounter data were performed using the R package nanostringr. The data generated underwent a series of normalizations to allow subsequent analyses.

**Deconvolution analyses**

Deconvolution analyses were performed using CIBERSORTx (1). CIBERSORTx is a method for characterizing cell composition of complex tissues from their bulk gene expression profiles, enabling large-scale analysis of cellular biomarkers and therapeutic targets. In our study it was used in order to identify the types of immune cells present in the FL tumor microenvironment (TME). Its application on gene expression data allowed the calculation of the relative cellular fractions of 22 immune cell types in the TME. To identify their differential expression between high- and low-risk groups according to the gene expression signature, Mann Whitney statistical analysis was performed.

**Image analyses**

For each slide, a representative region of interest was captured at 10x resolution (corresponding to a total area of 2448 x 1920 pixels) by using an Olympus BX53 light microscope with an Olympus DP27 camera. Regions with residual normal tissue and/or technical artifacts were excluded. The optimal color deconvolution was obtained by setting stain vectors using small regions of interest representing hematoxylin, Fast Red® (FOXP3), and background. Cells were detected using the watershed algorithm-based Detect Cells tool, using the optical density sum to segment nuclei with a cytoplasmic expansion of 2 µm; nuclei with an area < 10 μm^2^ or > 400 μm^2^ were automatically discarded. After segmentation, an expert hematopatologist (VT) manually quality-checked all outputs and removed areas with non-cellular overstaining artefacts and mis-segmented nuclear detections. Cells were classified as “FOXP3-positive” with the Simple Classifier tool, using the nucleus optical density mean of Fast Red® and manually setting the positive threshold after a human quality-check. The percentage of FOXP3-positive cells was subsequently calculated by dividing the count of positive cells by the count of total cells.

**SUPPLEMENTARY TABLES**

| **Clinical Trial (N° patients)** | **Procedure for determining the response** |
| --- | --- |
| *SAAK 35/98 (N=7)* | Computer tomography (CT) scans at 12 weeks, 7, 12, 18, 24 months;  Then yearly or sooner when clinically required. (2) |
| *SAAK 35/03 (N=52)* | CT scans at 11-13 weeks after start of induction therapy.  Then every 6 months during first 5 years (or until occurrence of event). (3)  IWG response criteria for NHL. |
| *SAAK 35/10 (N=9)* | CT scans at 10 (±1), 23 (±1) weeks, 30 months and 5 years. (4)  IWG response criteria for NHL. |
| *PF-05280586 (N=5)* | CT or fluoro-deoxy-glucose positron emission tomography (FDG-PET) scans at the end of therapy, every 3 months for 1 year; then ultrasound every 3 months. (5)  Revised response criteria for malignant lymphoma. (6) |
| *JASMINE (N=4)* | CT scans at 12 and 28 weeks. Then ultrasound every 3 months for the first year, every 6 months up to 5 years and once a year until progression. (7)  IWG response criteria for NHL. |
| *OFF-LABEL RITUXIMAB (N=4)* | Response criteria for malignant lymphoma. (8)  Ultrasound every 3 months for the first year, every 6 months up to 5 years and once a year until progression. |

**Supp. Table 1.** *Restaging procedures specified in the clinical trials considered in the study and used to assess patients’ responses.*

**REFERENCES**

1. Newman AM, Steen CB, Liu CL, Gentles AJ, Chaudhuri AA, Scherer F, et al. Determining cell type abundance and expression from bulk tissues with digital cytometry. Nat Biotechnol. 2019 Jul;37(7):773–82.

2. Martinelli G, Hsu Schmitz SF, Utiger U, Cerny T, Hess U, Bassi S, et al. Long-Term Follow-Up of Patients With Follicular Lymphoma Receiving Single-Agent Rituximab at Two Different Schedules in Trial SAKK 35/98. JCO. 2010 Oct 10;28(29):4480–4.

3. Taverna C, Martinelli G, Hitz F, Mingrone W, Pabst T, Cevreska L, et al. Rituximab Maintenance for a Maximum of 5 Years After Single-Agent Rituximab Induction in Follicular Lymphoma: Results of the Randomized Controlled Phase III Trial SAKK 35/03. JCO. 2016 Feb 10;34(5):495–500.

4. Zucca E, Rondeau S, Vanazzi A, Østenstad B, Mey UJM, Rauch D, et al. Short regimen of rituximab plus lenalidomide in follicular lymphoma patients in need of first-line therapy. Blood. 2019 Jul 25;134(4):353–62.

5. Sharman JP, Liberati AM, Ishizawa K, Khan T, Robbins J, Alcasid A, et al. A Randomized, Double-Blind, Efficacy and Safety Study of PF-05280586 (a Rituximab Biosimilar) Compared with Rituximab Reference Product (MabThera®) in Subjects with Previously Untreated CD20-Positive, Low-Tumor-Burden Follicular Lymphoma (LTB-FL). BioDrugs. 2020 Apr;34(2):171–81.

6. Cheson BD, Pfistner B, Juweid ME, Gascoyne RD, Specht L, Horning SJ, et al. Revised Response Criteria for Malignant Lymphoma. JCO. 2007 Feb 10;25(5):579–86.

7. Niederwieser D, Hamm C, Cobb P, Mo M, Forsyth C, Tucci A, et al. Efficacy and Safety of ABP 798: Results from the JASMINE Trial in Patients with Follicular Lymphoma in Comparison with Rituximab Reference Product. Targ Oncol. 2020 Oct;15(5):599–611.

8. Cheson BD, Fisher RI, Barrington SF, Cavalli F, Schwartz LH, Zucca E, et al. Recommendations for Initial Evaluation, Staging, and Response Assessment of Hodgkin and Non-Hodgkin Lymphoma: The Lugano Classification. JCO. 2014 Sep 20;32(27):3059–67.
